# The Creation of a Practice-Based Network of Pharmacists Working in Family Medicine Groups (FMG)

**DOI:** 10.3390/pharmacy7030108

**Published:** 2019-08-05

**Authors:** Anne Maheu, Marie-Claude Vanier, Léonie Rouleau, Nicolas Dugré, Line Guénette

**Affiliations:** 1 Faculty of Pharmacy, University of Montreal, Montreal, QC H3T 1J4, Canada; 2 Centre Intégré Universitaire de Santé et de Services Sociaux (CIUSSS) du Nord-de-l’île-de-Montréal, GMF-U Bordeaux-Cartierville, Montreal, QC H3M 2X6, Canada; 3 Centre Intégré de Santé et de Services Sociaux (CISSS) de Laval, GMF-U Cité-de-la-Santé de Laval, Montreal, QC H7M 3S7, Canada; 4 Centre Intégré Universitaire de Santé et de Services Sociaux (CIUSSS) du Nord-de-l’île-de-Montréal, GMF-U Sacré-Cœur, Montreal, QC H3M 3A9, Canada; 5 CHU de Québec-Université Laval Research Centre, Population Health and Optimal Health Practices Research Unit, Quebec, QC G1S 4L8, Canada; 6 Faculty of Pharmacy, Laval University, Quebec, QC G1V 0A6, Canada; 7 Centre Intégré Universitaire de Santé et de Services Sociaux (CIUSSS) de la Capitale-Nationale, Quebec, QC G1C 3S2, Canada

**Keywords:** pharmacist, clinical pharmacy, primary care, practice-based research network, professional practice, intra-professional collaboration, inter-professional collaboration

## Abstract

A needs assessment study of pharmacists working in family medicine groups (FMG) demonstrated the necessity to build a practice-based network. This network would foster a faster integration into FMG and a more efficient collaborative practice. It would also take advantage of an existing practice-based research network (PBRN)—the STAT (*Soutien Technologique pour l’Application et le Transfert des pratiques novatrices en pharmacie*) network. A working group of nine FMG pharmacists from the different regions of the province of Quebec, Canada, and a committee of partners, including the key pharmacy organizations, were created. Between January 2018 and May 2019, nine meetings took place to discuss the needs assessment results and deploy an action plan. The practice-based network first year activities allowed identifying pharmacists working in FMGs across the province. A directory of these pharmacists was published on the STAT network. The vision, mission, mandate, name («*Réseau Québécois des Pharmaciens GMF*») and logo were developed. The first few activities include: Bi-monthly newsletters; a mentorship program; short evidence-based therapeutic letters (pharmacotherapeutic capsules) and a start-up kit to facilitate integration of these pharmacists. The Quebec FMG pharmacist practice-based network has been launched. It is planned to evaluate the members’ satisfaction in late Spring 2020 with regards to activities and resources provided.

## 1. Introduction

In most Canadian provinces, pharmacists are now members of primary healthcare teams [1], such as family medicine groups (FMGs). FMG pharmacists provide direct patient care in collaboration with other healthcare professionals. They also support the FMG team to optimize patients’ pharmacotherapy. In Ontario, ten years after inclusion of pharmacists in these clinics, 111 (58%) family health teams had one or several pharmacists in their team [2]. The revision of the primary care framework by the Quebec Ministry of Health and Social Services, in 2015, has resulted in a broad integration of pharmacists in FMGs. A small proportion of FMG pharmacists work in university-affiliated FMGs (FMG-U) and are therefore also involved in teaching family medicine residents. A few have integrated super clinics (FMG-R) that provide increased access (i.e., they are open seven days a week, 12 h per day) to a broader range of primary care services for semi-urgent and urgent needs. According to the Quebec Ministry of Health, 79% of the 333 FMGs (FMG, FMG-U and FMG-R) had a working agreement with one or more pharmacists in January 2019 [3].

Practicing in a primary healthcare team with other healthcare professionals has been shown to be challenging for pharmacists [4,5]. Participating in a practice-based network can ease integration and provide support [6]. In 2018, we surveyed 178 FMG pharmacists to assess their needs and found that developing specific training, providing mentorship and disseminating information about their role to other healthcare professionals were among their most critical needs [7,8,9]. Publishing a contact list of all FMG pharmacists and their work location was also identified as a key priority to facilitate intra-professional collaboration as well as networking between FMG pharmacists [10]. Furthermore, we found most FMG pharmacists were self-employed and worked by themselves. Therefore, building a practice-based network was foreseen as a solution to interconnect them and provide them with tools and training required to optimize their integration and their practice.

The STAT (*Soutien Technologique pour l’Application et le Transfert des pratiques novatrices en pharmacie*) network is an existing practice-based research network (PBRN) for primary care pharmacists. Its web-based platform (www.reseaustat.ca) was launched in 2014 and there were 1233 members in April 2018. It is free to register. Our research team hypothesized that building a specific FMG pharmacists’ practice-based network could help increase participation and engagement of the STAT network members [11]. Besides, the web-based platform could facilitate networking and serve as a location to deposit practice tools and documents. This article describes approaches used to create and operationalize this new practice-based network for FMG pharmacists and discuss some of its impact on the STAT network members’ engagement.

## 2. Materials and Methods

Our research initiative used findings from the needs assessment [7,8,9] combined with qualitative discussions with a FMG pharmacist working group and an advisory committee of key organizations and partners (below). Collaborations were established with knowledge transfer experts, network and primary care researchers (including: “Strategy for Patient-Oriented Research” unit group (“*Unité soutien SRAP*"), Primary care practice-based research network (“RRAPPL”)). A structure for an “*Action Plan*” was developed and agreed upon with the researchers (see Table 1).

A working group of nine pharmacists from the different regions of the province of Quebec and with different level of experience working in a FMG was created in early 2018. Individuals were selected to ensure a good representation from different practice settings (FMG, FMG-U and FMG-R) and different type of working agreements (self-employed vs employment with the regional health authorities). The working group mandate was to: (1) Pre-test and comment on the questionnaires for the needs assessment survey; (2) contribute to interpretation and prioritization of the survey results; (3) participate in action plan development. The working group also provided regional insights on issues and initiatives and helped identify regional leaders and exchange groups. During the first 15 months of its creation, seven formal meetings took place either in person, by teleconference or visioconference. In addition, frequent email communications provided ongoing updates to the group. Individual working group members were and continue to be involved in projects to implement the action plan.

**Figure 1:**
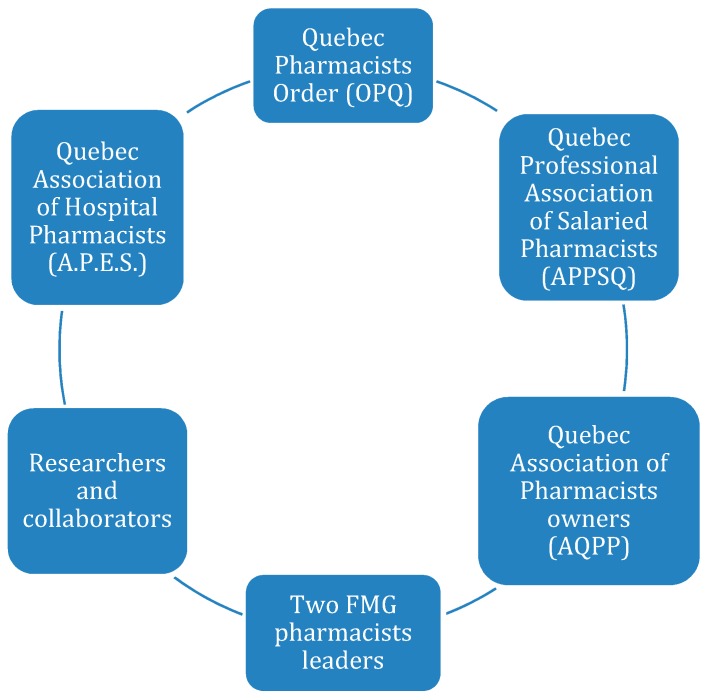
Advisory committee.

A committee of partners including the key organizations interested in the practice of pharmacists in FMG was established. The partners composing the advisory committee are detailed in Figure 1. The group was established to provide strategic direction and ensure alignment with activities for FMG pharmacists within their respective organization. They were consulted early on to provide guidance on needs assessment questionnaires and to discuss identification and recruitment of FMG pharmacists. Once the needs assessment was completed, the committee commented and challenged the content and prioritization of the action plan developed by the working group and made recommendations for the working group to consider. Between January 2018 and May 2019, two meetings took place with the advisory committee and frequent email communications provided ongoing updates to the advisory committee members.

**Figure 2:**
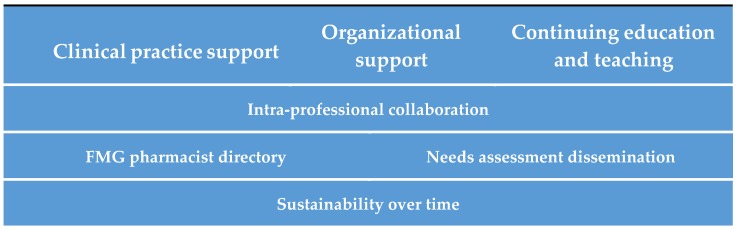
Themes of the Action Plan.

**Figure 3:**
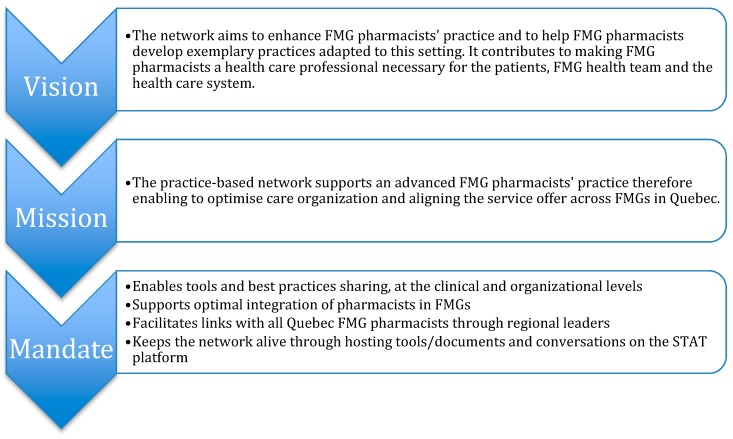
Vision, mission and mandate of the FMG pharmacists’ practice-based network.

The survey results (which have been published elsewhere [7,8,9]) were used to build the action plan that was co-developed with the working group and discussed with the advisory committee. Actions that should first be deployed (the “quick win”) to have an immediate impact were identified. The other action items were organized into broad themes (Figure 2) and prioritized looking at the degree of perceived importance, resources required and challenges to address them using a three-level priority (P1: Most important action to prioritize to P3: Action requiring significant resources for completion or with a long-term horizon). In parallel to writing the action plan, we developed the vision, the mission and the mandate of the practice-based network (Figure 3).

We undertook to identify all pharmacists working in FMGs to invite them to participate in the practice-based network. Their recruitment was initiated through phone calls made by five pharmacy students to all of Quebec FMGs in the spring of 2018. Other activities to identify and reach out to FMG pharmacists included publicity at different Quebec pharmacy conferences, email announcements by organizations from the advisory committee and through social media. A link to a “Google doc” was available for FMG pharmacists to register to the practice-based network. Word of mouth between FMG pharmacists has also enable the continued identification and registration of FMG pharmacists new to the position.

## 3. Results

It was clear, from the needs assessment results, that a practice-based network would address many of the issues raised by the respondents and facilitate FMG pharmacists’ practice and promote a more consistent role for these pharmacists across the province. In addition, it was expected that FMG pharmacists would stimulate participation on the STAT network platform. At the time of writing this article, 342 FMG pharmacists have been identified as such and invited to join the practice-based network in the province of Quebec and 284 have registered.

### 3.1. Characteristics of FMG Pharmacists and Their Work Settings

Characteristics of the 178 (60%) FMG pharmacists who completed the needs assessment survey have been described in detail elsewhere [7,9]. In summary, most are women (71.9%) younger than 40 years old (71.9%). Most are self-employed (60.7%) and also work as community pharmacists (76.4%). On average, they work in a FMG for 11.2 h a week (range 1–36; standard deviation [SD]: 6.8). With regards to FMGs, most of them are not affiliated with a university (66.3%) and most hire only one pharmacist (48.3%). The average pharmacists’ hours per FMG (combining for all the pharmacists working in the FMG) is 16.3 h a week (range 3–48; SD: 6.8).

### 3.2. Action Plan

The “quick win” initiatives from the action plan include for 2018–2019: Building a directory, launching newsletters, implementing a mentorship program, developing a communication plan, a starter kit and short-evidence-based therapeutic letters (pharmacotherapeutic capsules). They are described in details below. Longer-term activities or those requiring more resources or consultations are also included in the action plan but their completion will span over the next two to five years depending on the resources and ongoing re-prioritization.

### 3.3. Directory

Publication of a FMG pharmacists’ directory in the summer of 2018 was the first milestone accomplished by the practice-based network. An updated version is uploaded on a quarterly basis on the STAT network (www.ReseauSTAT.ca) platform on a special tab that was created for easy retrieval. The directory includes the name and address of the FMG clinic and regional health authority, the FMG pharmacist’ professional contact information (name, email and telephone), area of interest/specialization for the FMG pharmacist and the EMR (electronic medical record) system used. The publication of the directory was communicated on relevant social media and in bulletins of advisory committee organizations. As of June 1st 2019, the directory has been viewed 1234 times and downloaded 557 times since it was first published.

### 3.4. Network Logo and Name

**Figure 4:**
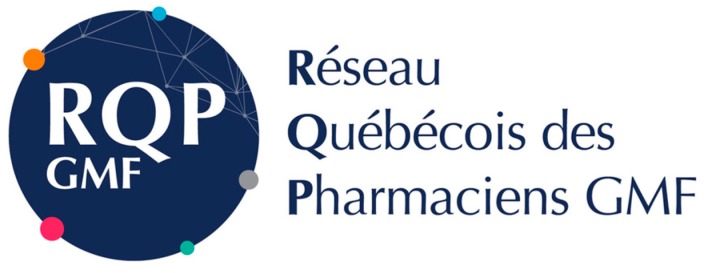
Name and logo of the Quebec network of FMG pharmacists.

We felt it was important to build a strong identity to the practice-based network and after several brainstorming sessions with the working group, a logo and a name were selected (Figure 4). The “*Réseau Québécois des Pharmaciens GMF—RQP GMF*” (Quebec network of FMG pharmacists) was created.

### 3.5. Newsletters

Newsletters were launched in December 2018 with the network name and logo. The “mailchimp” framework was used and the objective is to send a newsletter every 2 to 3 weeks with fresh and interesting information. Eleven general mailings have been done in the six months following its introduction. New programs or tools uploaded on the STAT network are highlighted in the newsletter to prompt network activities. Distinct newsletters are also customized and sent to FMG pharmacists involved in specific projects. In addition to the FMG pharmacists registered to the network (n = 284), 96 individuals have requested to receive these newsletters. The individuals are mostly stakeholders interested in FMG pharmacy practice or pharmacists wanting to join this novel type of practice.

### 3.6. Mentorship Program

A structured mentorship program was introduced in February 2019. It took six months to develop and was launched with online training to enable participants to learn the basics of mentorship. Twenty-five (25) pairs of mentor-mentee were put together. A few mentors agreed to have multiple mentees to accommodate all the mentees’ requests. The program is of one-year duration. Suggestions are made with regards to the frequency of meetings and a proposed structure for discussion at each time point is provided. Program evaluation is planned in the first quarter of 2020 to assess usefulness and impact as well as changes that should be made if a phase two is deployed.

### 3.7. Communication Plan, Starter Kit and Practice Tools

Promoting the role of FMG pharmacists internally (to other healthcare professionals of each FMG) and externally (to community pharmacists, regional health authorities, physicians’ organizations, etc.) was identified as a priority. The working group developed a communication plan while the researchers worked on knowledge translation activities aiming at publicizing the role of FMG pharmacists. First, initiatives to be implemented were identified and included in the action plan. Second, several presentations of the needs assessment survey, including activities performed by FMG pharmacists, were made at pharmacists’ and primary care conferences. Third, tools to be used by FMG pharmacists to promote their role were developed. Two fourth-year pharmacy students developed PowerPoint presentations and a two-page visual document targeting different audiences as part of a one-month internship with research team members. These tools were validated by the working group, disseminated through the newsletters and uploaded on the STAT network platform. These documents had been viewed a total of 552 times and downloaded 176 times two months after becoming available. Finally, a starter kit for FMG pharmacists new to the practice was developed by a team of five fourth-year pharmacy students under the supervision of the research team and working group members. The starter kit includes 10 key steps facilitating a fast and effective integration into the FMG. It also contains practical tips as well as links to relevant documents. Their internship enabled to test and to refine the starter kit with 28 FMG pharmacists. The final version of these tools is available on the STAT network platform.

### 3.8. Short Evidence-Based Therapeutic Letters (Pharmacotherapeutic Capsules)

Writing internal scientific communications or doing formal/informal education to physicians/FMG team members were frequent activities captured in the needs’ assessment survey. The working group felt that efforts and resources could be pooled to collectively develop pharmacotherapeutic capsules that would benefit members of the RQP GMF network. Indeed, FMG pharmacists could use the information to optimize their practice and share it within their respective FMG. Efficiency would be gained and practice standardization between FMGs could be reached. An editorial team was put in place and 44 network members accepted to contribute to writing capsules. Consultations with knowledge translation experts led to the development of brief but frequent capsules, i.e., bi-weekly publications [12,13]. A name (“*InPHARMation*”) was given and branding of the bulletins was developed. The pharmacotherapeutic capsules will be stored on the two Quebec province’s Faculties of Pharmacy (Université de Montréal and Université Laval) websites.

Prior to launching these capsules, four demos were sent to the network participants in April 2019 to collect feedback on the structure, content and overall concept. The official start is scheduled in the summer of 2019. The newsletters will be used to share and promote the capsules with the FMG pharmacists and will include links to both universities websites for easy retrieval over time. Program evaluation is planned in 2020 to assess usefulness and impact as well as identify areas for improvement.

### 3.9. Long-Term Activities

Long-term activities that need a greater level of consultations to elaborate, require significant resources for completion or have a long-term horizon include: (1) Liaison with regional leaders and regional groups (such as the “regional pharmaceutical services committee” (called “CRSP”); (2) establishing collaboration with practice networks of FMG nurses and FMG social workers; (3) developing a structure to move from a research initiative to a real-life model for long-term viability of the network.

### 3.10. STAT Network—The Platform

The STAT network platform counted on 1233 registered participants when the initiative for creating the FMG pharmacists practice network was launched in the spring of 2018. Thirteen months later, the number grew to 1603 (370 new members (+30%) vs. 208 (+20%) in the previous year). Improvements to the platform have been made and a “specific area” for FMG pharmacists has been added to deposit the starter kit. Specific tabs were added in the library and in the forum section. Several documents have been deposited in the library in addition to the directory and tools to promote the role of the FMG pharmacists (mentioned above). Indeed, a very popular item was the toolkit of key references and websites (to add to a navigator). This toolkit was viewed 659 times and downloaded 412 times generating traffic on the platform not only by the FMG pharmacists but also by other pharmacists members of the STAT network. Seven FMG pharmacists’ conversations were held in the forum section.

## 4. Discussion

To our knowledge, the RQP GMF network will be one of the first focusing on family medicine group pharmacists’ practice. The practice-based network was developed using a thorough needs assessment and in partnership with a working group of FMG pharmacists’ representatives and an advisory committee of key organizations. This approach ensured a sound methodology while allowing for a rapid impact of the network activities. It enabled building from a solid ground of information while mobilizing interested individuals and stakeholders. The action plan has allowed identification of key priorities (“quick win”) for quick and impactful results. Initiatives supporting the network have started in December 2018, only three months following the completion of the needs assessment survey.

Our hypotheses that FMG pharmacists’ participation would lead to greater engagement and participation on the STAT network platform is supported by utilization data showing acceleration of registration of new members vs. the previous year (difference of 10%). Daily visits also increased, probably as a result of specific documents for FMG pharmacists being uploaded on the platform in addition to tools/documents that could be utilized by all pharmacists such as the toolkit of key references in primary care. Opportunities for other pharmacists to use tools/documents developed for FMG pharmacists are highly valuable to advance the pharmacy profession. The library remains a very popular destination on the STAT network platform. However, further research is needed to confirm whether this increased utilization is linked to the RQP GMF network.

The strong participation of the FMG pharmacists to the network activities such as the mentorship program, the pharmacotherapeutic capsules and the evaluation of the starter kit demonstrate interest in learning, exchanging and contributing to the practice-based network. It is noteworthy that the documents to support promoting the role of FMG pharmacists have been downloaded 176 times in a very short period of time. This highlights importance for FMG pharmacists to share information about their role with other team members and confirm the key priority status observed for this in the needs’ assessment survey.

Creating exchanges on the STAT network platform forum remains a challenge. Our personal experiences indicate that exchanges among FMG pharmacists often take place on social media (the Facebook group for FMG pharmacists) likely because it is easy to access and ideal for quick answers. Our vision was to move conversations of more complex issues, such as discussing patients’ cases, to the STAT network forum as the platform allows for connecting documents deposited in the library and easier retrieval of conversations. We will continue to explore whether there is a need for a forum for FMG pharmacists on the STAT network platform and the best format to ensure relevance.

Moving from a research initiative to a sustainable structure remains a significant issue and one of the biggest challenges for the RQP GMF. Conversations have been initiated with regional pharmacy committees (the “regional pharmaceutical services committee” (CRSP)), the Ministry of Health and Social Services and the primary care practice university Institutes to explore options. A mid-term solution we are looking at is obtaining a grant to maintain activities until a permanent approach is secured.

In a recent article, Gillespie [2] mentions that in Ontario there has been networking and education through a volunteer-run FMG pharmacist network. However, no specific information was found in the literature on that initiative. Many Canadian FMG pharmacists are members of the Primary Care Pharmacists Pharmacy Specialty Network (PSN) that is jointly supported by the Canadian Pharmacists Association (CPhA) and the Canadian Society for Hospital Pharmacists (CSHP) [14]. Exchanges are supported via an email “listserv” mechanism and 464 pharmacists are registered to the listserv. The PSN shares practice-based resources, provides a forum for posing clinical questions and eliciting case feedback, and discusses how to advocate for the role and practice development of pharmacists in the primary care setting. A few Quebec FMG pharmacists are members of that PSN, especially those coming from the academic centers. Low adherence to the Canadian PSN by Quebec FMG pharmacists might be linked, in part, to language barrier since the majority of Quebec pharmacists are francophone. Indeed, they might not feel comfortable to discuss complex issues in English or may look for French version of clinical tools. This reinforces relevance of a lively community of practice on French language platform.

In the United States, Dickerson [15] published in 2007 the characteristics and activities of 81 FMG pharmacists, from 48 sites, joining a PBRN in primary care setting. However, this network, from the American College of Clinical Pharmacy Network, was geared at academic pharmacists working in primary care setting and providing advanced care in order to develop research on clinical practice in primary care setting. This type of network would likely be of interest only to Quebec FMG pharmacists working in University affiliated family medicine teaching clinics. At its current stage of development, our community of practice needs to focus mainly on supporting pharmacists’ good integration in a novel practice and consolidation of optimal and consistent pharmaceutical care throughout the province FMGs. The level of integration reached by pharmacists in family medicine clinics has been correlated in several papers with optimal patient outcomes and pharmacy benefits [4,5,6,16]. Our practice-based network has thus initially prioritized practical interventions to promote and facilitate the full integration of FMG pharmacists into their FMG settings. However, as a second phase development, a branch of our practice-based network could possibly evolve towards a PBRN since nearly 25% of the FMG pharmacists are from academic centers. Other FMG pharmacists could participate in quality assessment or research initiatives and the PBRN could facilitate initiating, exchanging and disseminating those initiatives.

## 5. Conclusions

The Quebec FMG pharmacists’ practice-based network has been launched. Several activities have been introduced shortly after the completion of the needs assessment and the level of participation by FMG pharmacists is high. The majority of FMG pharmacists have registered to the network and to the directory, which is available on the STAT network. The STAT network has benefited from the launch of the FMG pharmacists’ practice-based network with acceleration of registration of new members, greater engagement and participation of the members.

We strongly believe that our network will facilitate integration of FMG pharmacists into their FMG settings and lead to greater patient care and pharmacy benefits.

It is planned to evaluate the benefits of our practice-based network and satisfaction of the members with regards to activities and resources in 2020, a year after commencement of initiatives.

## Figures and Tables

**Table 1:** Structure of the Action Plan (with the “starter kit” as an example).

ACTION (#)	PROJECT (detailed sub-components)	STATUS (% completion)	CHAMPION (other collaborators)	LINK with other projects	PRIORITY LEVEL (P1, P2, P3)	KEY PERFORMANCE INDICATORS (KPIs)	TIMELINE (Beginning—End)	COMMENTS	STATUS (Green; yellow; red)
1.1	Starter Kit Project by pharmacy students («integrative internship» Development of starter kit concept Validation with working group Evaluation by 20 FMG pharmacists Publication on the STAT network	Done Done Done June 2019	Marie-Claude (Anne and Léonie)	- Role - Service offer	P1	- Project development 05/2019 - Evaluation by 20 FMG pharmacists (05/2019) - Publication 06/2019	10/2018–06/2019	- Tips and tricks for easier and faster integration - Service offer - Working with web developer for creating new FMG pharmacists’ corner on STAT network platform	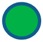
